# Silencing of the nucleocytoplasmic shuttling protein karyopherin a2 promotes cell-cycle arrest and apoptosis in glioblastoma multiforme

**DOI:** 10.18632/oncotarget.26033

**Published:** 2018-09-11

**Authors:** Ramon Martinez-Olivera, Angeliki Datsi, Maren Stallkamp, Manfred Köller, Isabelle Kohtz, Bogdan Pintea, Konstantinos Gousias

**Affiliations:** ^1^ Department of Neurosurgery and Neurotraumatology, BG University Hospital Bergmannsheil, 44789 Bochum, Germany; ^2^ Department of Laboratory for Neurosurgical Research, BG University Hospital Bergmannsheil, 44789 Bochum, Germany; ^3^ Institute for Transplantation Diagnostics and Cell Therapeutics, Heinrich-Heine University, 40225 Düsseldorf, Germany; ^4^ Medical School, Rheinische Friedrich-Wilhelms University of Bonn, 53121 Bonn, Germany; ^5^ Department of Surgical Research, BG University Hospital Bergmannsheil, 44789 Bochum, Germany; ^6^ Department of Neurosurgery, University Hospital of Marburg, 35033 Marburg, Germany

**Keywords:** glioblastoma multiforme, karyopherin a2, U87 MG, nucleocytoplasmic transport

## Abstract

We have previously shown that the nucleocytoplasmic carrier karyopherin a2 (KPNA2) is overexpressed in glioblastoma multiforme (GBM) whereas its expression is inversely associated with patient prognosis. However, the promoting role of KPNA2 in gliomagenesis is still poorly understood. This study aims to further elucidate this role of KPNA2 in *in vitro* GBM models.

From four different tested GBM cell lines, the U87MG showed the highest proliferation, low adherence and outgrowth in 3D clusters as well as the highest expression of KPNA2, all features conferring greater malignant behaviour. Silencing of KPNA2 via siRNA interference in those cells significantly decreased their proliferative capacity (*p* = 0.001). We further observed both a significant cell cycle phase arrest (*p* = 0.040) and the promoting of cellular apoptosis (*p* = 0.016) as well as a strong trend (*p* = 0.062) for an inhibition of nuclear import of c-Myc.

This study confirms that a higher expression of KPNA2 in GBM is associated with a more malignant phenotype also in *in vitro* models. While increased expression of KPNA2 promotes proliferation and survival of GBM tumour cells, silencing of KPNA2 conferred a less malignant behaviour. Our results strongly suggest that silencing of KPNA2 may play an important role in modulation of malignant features of GBM cells.

## INTRODUCTION

Increased nucleocytoplasmic shuttling has been linked to carcinogenesis in general [1], since a number of cell cycle regulators and transcription factors, both key mediators of tumorogenicity, are cumulatively translocated into the nucleus and therefor activated. The nucleocytoplasmic transport is mediated by a family of proteins, called karyopherins (importins and exportins), which have emerged as promising diagnostic and prognostic biomarkers in a variety of solid tumours [2]. A higher expression of the importin karyopherin a2 (KPNA2) in particular, has been associated with a poor prognosis of patients with pancreatic, breast, lung, prostate, colorectal, ovarian cancer as well as with brain tumours [3–10].

KPNA2 mediates the transport of macromolecules into the nucleus of cells upon binding to a specific recognition sequence, the nuclear localisation signal (NLS). The complex KPNA2/NLS/macromolecule is subsequently translocated through the nuclear pores into the nucleus. There, the transported macromolecule binds to the Ran-protein-guanosintriphosphat (Ran–GTP) and is released from KPNA2, which then returns into the cytoplasm [11].

KPNA2 is implicated in the malignant transformation of cells by regulating the DNA-repair proteins, the activation of apoptosis and the transport of tumour suppressors, transcription factors and oncogenes into the nucleus [3, 11–14]. Evidence for the role of KPNA2 in the tumorigenesis was provided by manipulating its expression. Diverse studies on animal tumour models could demonstrate that the proliferation rate of cancer cells increases significantly upon overexpression of KPNA2; in turn, the proliferation decreases upon KPNA2 antagonism [6, 15–17]. KPNA2 is also a main regulator of the cell cycle [16, 18]. A knockdown of KPNA2 resulted in a cell cycle phase arrest in adenocarcinoma cell lines of the lung- and ovarian- cancer [16, 19]. KPNA2 is also responsible for the translocation of important tumour associated molecules like BRCA1, p53, p27, APC, E2F1, Oct4, Nf-kB and c-Myc [16, 20, 21].

Noteworthy, Nf-kB, Oct-4 and c-Myc play a central role also in the genesis of glioblastomas (GBM, glioma WHO grade IV) [22–25]. Therefore, KPNA2 may additionally regulate gliomagenesis by mediating the nuclear import of above mentioned molecules. GBMs represent the most frequent primary malignant brain tumour, as they comprise 45% of them [26]. Despite the currently existing multimodal therapeutic modalities, mainly consisting of tumour resection combined with adjuvant radio- and chemotherapy, patients with GBM display a dismal prognosis of up to 15 months overall survival (OS) [27–31]. As previously mentioned, a higher expression of KPNA2 has been associated with a poorer OS and progression free survival (PFS) in a large series of patients with GBM [8]. However, the functional role of KPNA2 in genesis of GBM has not been elucidated yet.

Our study aims to analyse for the first time the oncogenetic role of KPNA2 in *in vitro* GBM models. After testing 4 different GBM cell lines (U118 MG; U87 MG; U138 MG; U373 MG), silencing of KPNA2 through siRNA interference will be employed to the cell line with the highest KPNA2 expression. The effect of KPNA2 silencing on cell morphology, proliferation activity, survival, apoptosis, cell cycle activity as well as the subcellular localisation of specific transcription factors will then be evaluated.

## RESULTS

**Figure 1:**
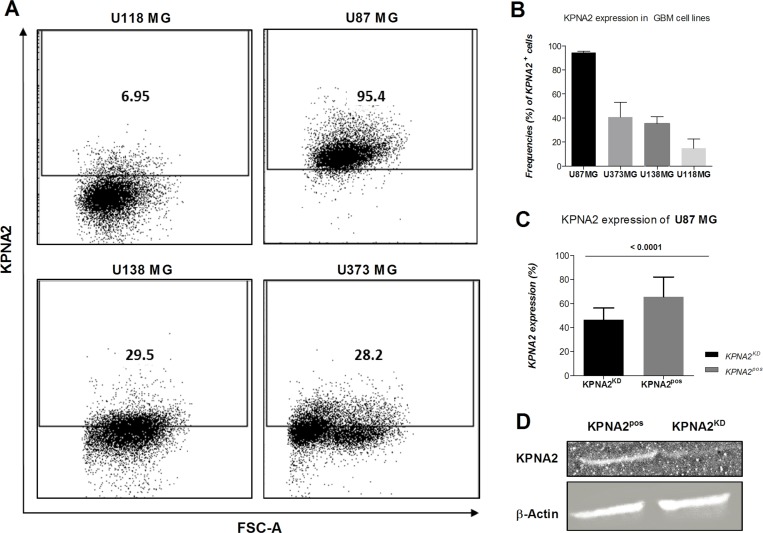
KPNA2 expression is overexpressed in the most aggressive GBM cell line U87 MG and significantly downregulated upon silencing of the importin (**A**) Flow cytometric analysis of intracellularly stained KPNA2 on the four different glioblastoma cell lines (U118 MG, U87 MG, U138 MG, U373 MG) shows highest expression of the importin in the cell line U87 MG. Intracellular staining was performed with the polyclonal antibody against KPNA2 (Santa Cruz; 1:50). (**B**) Quantification of the KPNA2 expression in the four different cell lines on protein level based on flow cytometry (*n* = 3). (**C**) Knock-down efficiency of KPNA2 after siRNA-interference is evaluated via intracellular immunofluorescence staining of KPNA2 in U87 MG cells showing a significant reduction of the importin based on total cell count (*p* < 0.001). (**D**) Knock-down efficiency of the siRNA was evaluated on protein level via western blot analysis in comparison to the housekeeping marker β-actin and confirmed downregulation of the KPNA2 protein expression. Actin expression was used as internal control and for normalization of protein expression levels. KPNA2^KD^:siRNA interfered.

Four different GBM cell lines (U118 MG; U87 MG; U138 MG; U373 MG) were analysed for their expression levels of the importin KPNA2, displaying the highest amounts in the cell line U87 MG as determined by flow cytometry (Figure 1A, 1B). These cell lines differ in their malignancy status based on their proliferative capacity, adhesion and migration behaviour. U87 MG is characterized as the most aggressive cell line, due to its high proliferation rates (as assessed by its division rate of 36 hr, data not shown) as well as its growth capacity in 3D clusters, and further showed the highest expression of KPNA2. Therefore, this cell line was utilized in this study to investigate the influence of the importin on tumour progression. Hence, KPNA2 was silenced via siRNA interference resulting in a significant reduction of the intracellular KPNA2 (*p* < 0.001). Expression levels were determined by immunofluorescence staining and western blot analysis in both the U87 MG cell line before (KPNA2^pos^) and after KPNA2 silencing (KPNA2^KD^) (Figure 1C, 1D).

**Figure 2:**
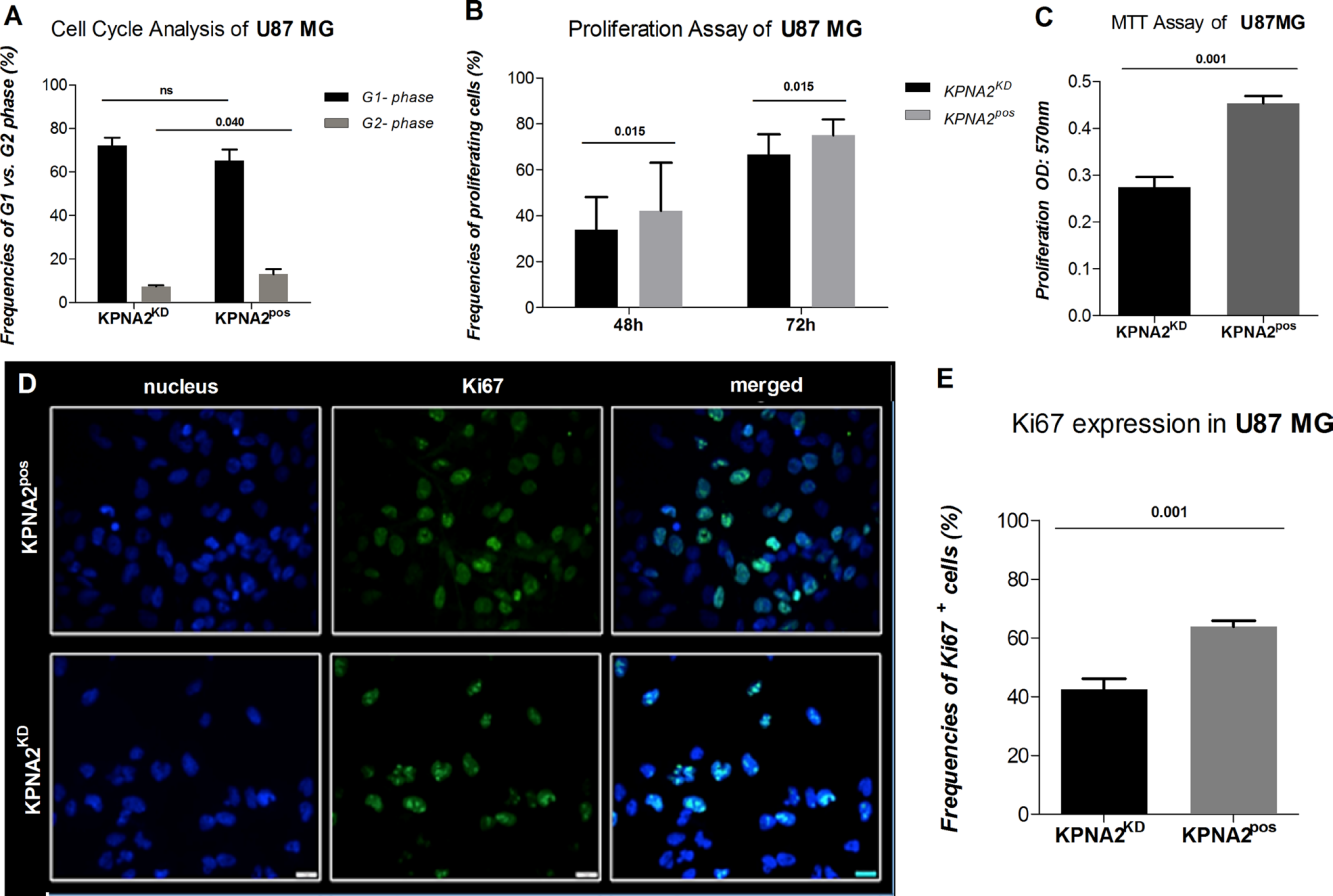
Silencing of KPNA2 is associated with cell-cycle phase arrest and decreased proliferation capacity of the cell line U87 MG (**A**) Cell Cycle analysis via flow cytometry displays a significant reduction of the cells detected in the G2-phase in the KPNA2^KD^ cells in comparison to KPNA2^pos^ (*p* = 0.040). Results are presented as frequencies of cells in the distinct phases of the cell cycle. (**B**) Proliferation of KPNA2^KD^ vs. KPNA2^pos^ cells is considerably inhibited after 48 h (*p* = 0.015) and 72 h (*p* = 0.015) of proliferation. Assays are performed with the CFSE-proliferation dye and analysed via flow cytometry (BD Calibur). (**C**) Proliferation of KPNA2^KD^ vs. KPNA2^pos^ cells is considerably inhibited after 48 h (*p* < 0.001) of proliferation. Assays are performed by the MTT-Assay (Roche) and analysed via BioTek ELISA-Reader. (**D**) Ki67 expression determined by intracellular immunofluorescence staining shows a significant reduction in expression levels after silencing of KPNA2. Nuclear staining was performed with DAPI. Ki67 (BD monoclonal MIB-1 antibody) with green fluorescence displays different proliferation states of the individual cells. Knockdown of KPNA2 reduces expression levels and alters the expression pattern of Ki67 in the siRNA treated cells. (**E**) Quantification of Ki67 expression displays meaningful reduction (*p* = 0.001) in the KPNA2^KD^ cells in comparison to the KPNA2^pos^ cells.

The importin KPNA2 has been described to play a crucial role in matters of the cell cycle and proliferation status in solid tumours of different origins. In brain tumours, however, its involvement is poorly understood up to date. Hence, cell cycle analysis was performed in both U87 MG KPNA2^KD^ and KPNA2^pos^ cells. A significant cell cycle phase arrest could be demonstrated as the G2 phase detected in KPNA2^KD^ cells was significantly reduced (*p* = 0.040) compared to their KPNA2^pos^ counterparts (Figure 2A; Supplementary Figure 1A). These findings align with the results obtained from a CFSE-proliferation analysis, where KPNA2^KD^ cells display a significant reduction in their proliferative capacity already after 48 h (*p* = 0.015) of observation in comparison to the KPNA2^pos^ cells (Figure 2B; Supplementary Figure 1B). Also, the proliferation potential of the two cell populations was determined by an MTT-assay, which reveals a significantly higher (*p* < 0.001) proliferative capacity of the KPNA2^pos^ cells, when compared to the KPNA2^KD^ cells (Figure 2C). In addition, KPNA2 silencing was associated with a significant reduction (*p* = 0.001) of the proliferation marker Ki67 in the KPNA2^KD^ population in comparison to their untreated control (Figure 2D, 2E).

The U87 MG cells displayed at their cell surface membranous protrusions, the so called filopodia, which have been already described before as marker for greater invasion and migration in GBM [32–34]. The silencing of KPNA2 with siRNA interference alters the cell morphology in terms of loss of the filopodia of the affected cells.

**Figure 3:**
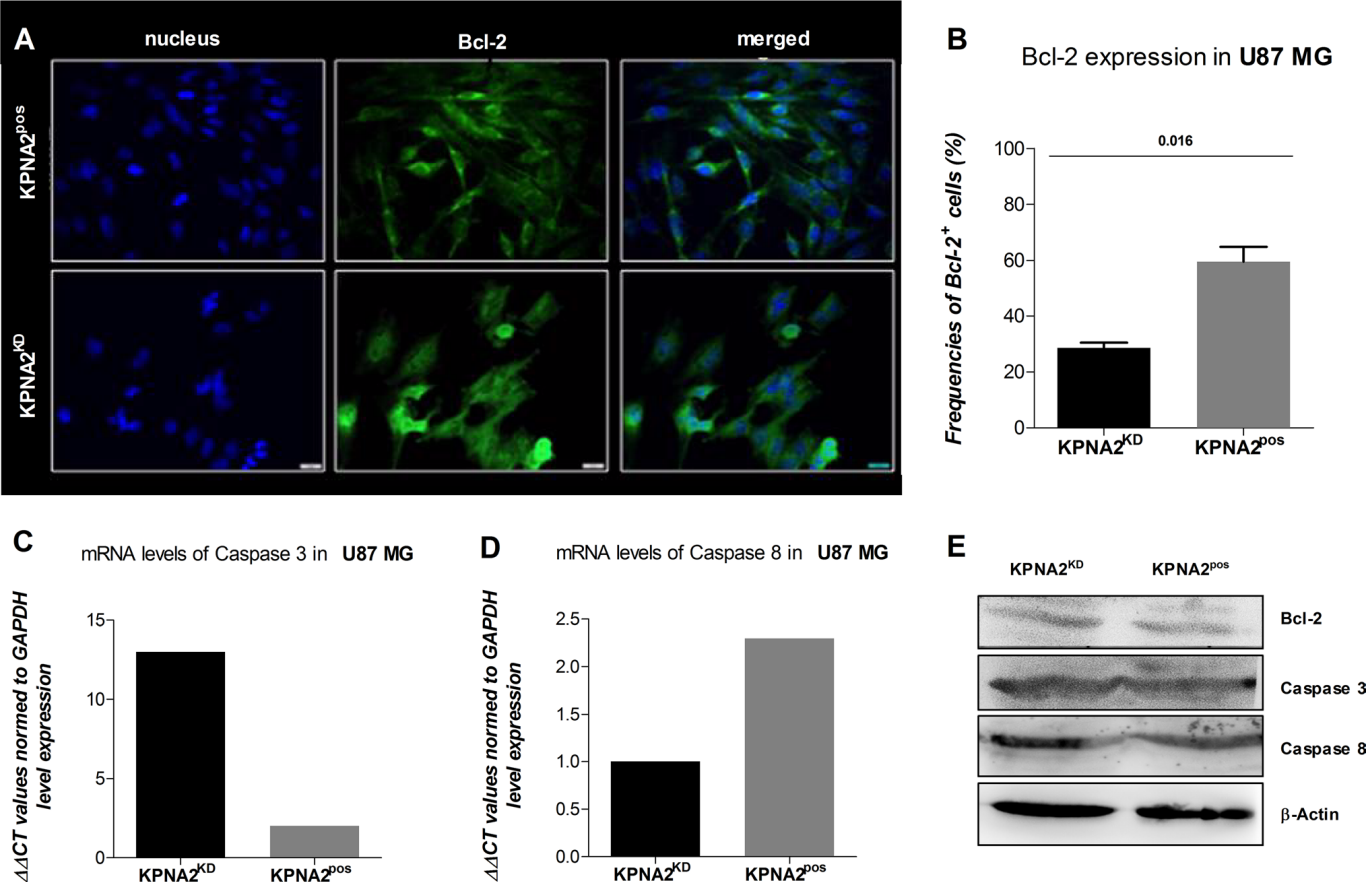
KPNA2 expression maintains survival and morphology of the GBM cell line U87 MG (**A**) The silencing of KPNA2 with siRNA interference alters the cell morphology of the U87 MG cells by reducing the filopodia of the affected cells. Bcl-2 expression (green) is expressed in KPNA2^KD^ vs. KPNA2^pos^ in significantly lower amounts based on relative expression of total cell counts, but different distribution patterns. While in the negative control the Bcl-2 molecules are detected closely and organized in the cell-body in close proximity to the nucleus, expression of Bcl-2 in the KPNA2^KD^ cells is diffuse and scattered. (**B**) Quantification of Bcl-2 expression displays a significant reduction (*p* = 0.016) in the KPNA2^KD^ cells in comparison to the KPNA2^pos^ cells. (**C** and **D**) Relative mRNA expression of caspase 3 and caspase 8 via quantitative Real-Time PCR reveals an up-regulation of the effector caspase 3 in the KPNA2^KD^ cells in comparison to the KPNA2^pos^ ones and a down-regulation of the initiator caspase 8 in the former. (**E**) The survival protein Bcl-2 and the apoptotic proteins caspase 3 and 8 were determined via western blot. Hereby, total protein is displayed with the normalizing loading control β-actin. No cleaved forms of either caspase 3 or caspase 8 were detected in KPNA2^pos^ nor in KPNA2^KD^ cells.

As KPNA2 is a nucleocytoplasmic shuttling receptor responsible for transporting several proteins from the cytoplasm to the nucleus, it might also be involved in mechanisms of cell survival and apoptosis. Hence, understanding the expression pattern and level of Bcl-2, an anti-apoptotic protein could give rise to the survival status of the cell line U87 MG after silencing KPNA2. While the KPNA2^pos^ cells display high expression levels of Bcl-2, mostly detected in close proximity to the cell nucleus, the KPNA2^KD^ cells show decreased Bcl-2 levels (*p* = 0.016) and more diffuse expression distributed over the whole cell body (Figure 3A, 3B).

Since Bcl-2 is known as an anti-apoptotic protein, we evaluated the initiation of programmed cell death after silencing of KPNA2 by analysing two main enzymes responsible for the apoptotic process on gene and protein level (Figure 3C–3E). Caspase 3 is known to be the effector-caspase when it comes to the execution of the programmed cell death and is normally present in every cell in an inactivated form. Specific detection of relative mRNA levels of the activated form of this enzyme shows high expression in the KPNA2^KD^ cells in comparison to their KPNA2^pos^ counterpart. Considering the initiator Caspase 8, which is responsible to activate the Caspase 3 and thereby changes its conformation, the relative expression levels of the two considered cell populations are reversed. Hence, Caspase 8 is significantly downregulated in the KPNA2^KD^ population after 48 h of siRNA interference.

**Figure 4:**
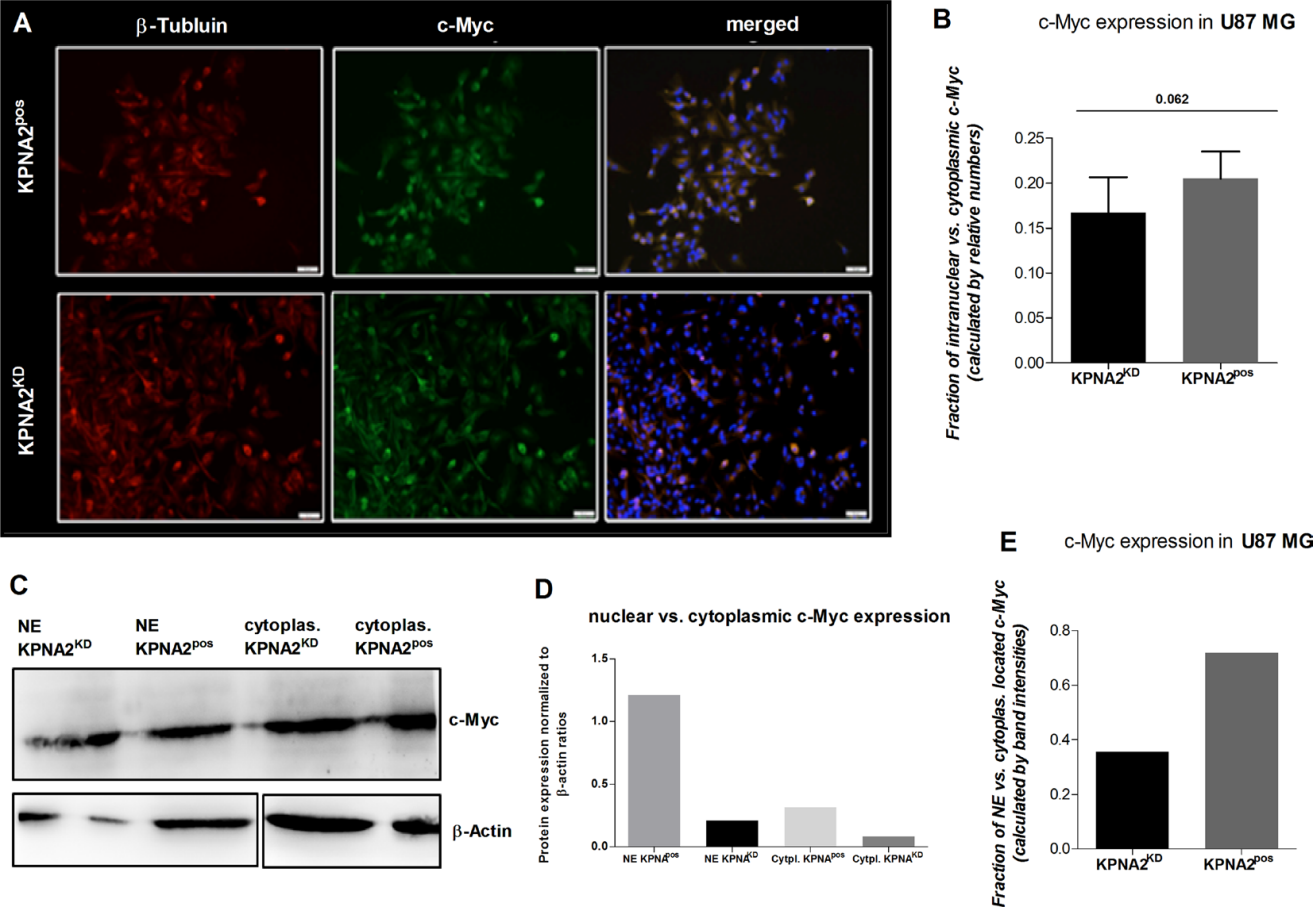
Silencing of KPNA2 alters the nuclear transport of the oncogene c-Myc in U87 MG cells (**A**) Immunofluorescence staining of the oncogenic transcription factor c-Myc shows expression of c-Myc in almost all cells of the U87 MG cell line regardless the treatment with or without siRNA targeting KPNA2. Co-staining with beta-Tubulin (red) for the cytoskeleton and DAPI (blue) for nuclear staining illustrates the subcellular localization of c-Myc (green). KPNA2^KD^ cells show less nuclear localization then KPNA2^pos^ cells. (**B**) Calculation of the relative distribution of nuclear/cytoplasmic c-Myc expression in KPNA2^pos^ and KPNA2^KD^ cells displays a strong trend towards a decreased nuclear expression of c-Myc in the KPNA2^KD^ cells (*p* = 0.062). (**C**)Western blot analysis of the nuclear vs. cytoplasmic fractions of KPNA2^KD^ and KPNA2^pos^ cells shows a lower c-Myc expression in the knockdown cells in the nuclear counterpart as well as in the cytoplasm when compared to both cell compartments of the negative controls. (**D**) Normalization of the c-Myc protein ratios to the corresponding β-actin loading controls shows the highest expression of c-Myc in the nucleus of KPNA2^pos^ cells, as expected. (**E**) Illustration of the ratio between nuclear vs. cytoplasmic c-Myc shows reduced nuclear c-Myc expression in the KPNA2^KD^ cells.

It is known that the oncogenic transcription factor c-Myc is dependent on nuclear transporter proteins like KPNA2 to be transported from the cytoplasm into the nucleus. In order to investigate the role of KPNA2 in the subcellular translocation of c-Myc also in GBM U87 MG cells, KPNA2^KD^ and KPNA2^pos^ cells were co-stained for c-Myc as well as for DAPI and beta-Tubulin for the visualisation of nucleus and the cytoskeleton, respectively. The immunofluorescence staining revealed >95 % c-Myc expression in both cell populations (Figure 4A). However, a further determination of the intracellular location of c-Myc showed a strong trend for a decreased nuclear expression of c-Myc in the KPNA2^KD^ population in comparison to its KPNA2^pos^ control (*p* = 0.062) (Figure 4B). The immunofluorescence results are confirmed by the determination of nuclear vs. cytoplasmic c-Myc of the different cell populations by analysing the two cell-compartments separately by western blot. By fractionizing the KPNA2^pos^ and KPNA2^KD^ cells, a clear analysis of both parts reveals a generally lower c-Myc expression of KPNA2^KD^ cells when normalized to β-actin, but also an overall higher expression of the transcription factor within the cytoplasmic fraction of the cells. Further, a direct comparison of the fraction-ratio confirms a higher ratio for KPNA2^pos^ cells (Figure 4C–4E). Thus, c-Myc may predominantly reside in the cytoplasm after silencing of KPNA2 and remain inactive.

Within this study, also further transcription factors relevant for oncogenic processes and maintenance of stem cell like character (Oct-4) or anti-apoptotic proteins, like NF-kB, have been evaluated for their dependency on KPNA2 in regards to their nuclear transportation. Even though a trend towards a reduced accumulation of the TFs in the nucleus after silencing of KPNA2 could be detected, the results are not significant (data not shown).

## DISCUSSION

Despite current advances in diagnostic and therapy strategies, patients with GBMs still show a poor prognosis [27–30]. Even after the establishment of a more individualised therapy based on clinical characteristics (such as age, Karnofsky score) but mainly on molecular profiles, only a slight increase of OS has been observed [30, 31]. Molecular milestones such as the status of the O6-Methlyguanin-DNA-Methyltransferase (MGMT), the Isocitrat-dehydrogenase 1 and 2 (IDH 1- und 2) as well as the 1p/19q status [29, 35] have contribute to identify patients with potential better outcomes. However, since these markers may not be regulated in favour of the patients yet.

Prognostic markers which could be modulated in order to prolong the survival of patients with GBM are needed. The importin KPNA2 may represent an optimal candidate for this scope since it may have prognostic value in predicting OS and PFS of patients with GBMs, as previously shown [8]. Moreover, KPNA2 is a molecule which could be antagonized permitting therapeutic intervention in cancer models. Manipulation of KPNA2 expression has been successfully performed *in vivo* and *in vitro* [7, 16, 36]. However, the involvement of KPNA2 in the pathogenesis of GBM has not been studied *in vitro* yet.

In the current study we investigated for the first time the promoting role of KPNA2 in the pathogenesis of GBM in four different GBM cell lines ((U118 MG; U87 MG; U138 MG; U373 MG). The cell line U87 MG displayed more malignant patterns compared to the remaining cell lines, in terms of higher proliferation rates (division time 36 hr) and outgrowth in 3D clusters. In accordance with our previous results, which identified a higher expression of KPNA2 in patients with a higher WHO grade of malignancy in a large series of patients with cerebral astrocytomas our present *in vitro* analysis KPNA2 confirmed KPNA2 as marker of malignancy as it was predominantly detected in the most aggressive U87 MG GBM cell line. The cell line U87 MG was subsequently chosen for our further *in vitro* analysis as it showed the highest KPNA2 expression as well as the best KPNA2 suppression through siRNA interference.

Several studies have identified KPNA2 as a promoter of proliferation in a number of tumour cell lines, whereas the suppression of KPNA2 expression resulted in an anti-proliferative activity [12, 15, 16]. Similarly, in the present study KPNA2^pos^ cells showed a significant higher proliferation capacity compared to the KPNA^KD^ cells. Noteworthy, the proliferation potential has been tested via both CFSE-proliferation analysis, MTT assay and immunohistochemically detection of the proliferative protein Ki67. Thus, KPNA2 may promote proliferation in GBM cells as well.

The direct microscopic analysis of a tumor cell line, i.e evaluation of the cell line adhesion, outgrowth, migration and morphology in general, may reflect its malignant potential. Membranous protrusions of the cell surface of tumor cells have been described in GBM, in particular [32]. These micro-spikes, called also filopodia demonstrate high infiltrative potential of GBM; the loss of them is associated with reduced migration and invasion potential of the cells [32–34]. In our study the suppression of KPNA2 through siRNA interference conferred a less malignant morphology to the U87MG cell line. The characteristic aggressive and infiltrative outgrowth of the above cell line mainly based on the activity of their filopodia, has been altered by silencing of KPNA2 since in such manner we observed a loss of filopodia of the affected cells. A potential reason for this de-formation of the cell body of U87MG KPNA2^KD^ cells might be the diminished shuttling of the S100A6 protein. S100A6 depends on KPNA2 to be translocated into the nucleus, where it actively participates in processes of cell formation [37].

Cell cycle phase arrest upon silencing of KPNA2 has been shown before in human cancers outside the CNS. Huang *et al.* and Yang *et al.* induced cell cycle phase arrest in epithelial ovarian carcinoma cells and human hepatocellular carcinoma cell lines HepG2 and SMMC-7721 after suppressing KPNA2 through siRNA interference, respectively [16, 36]. The current study identified a significantly reduced proportion of cells in the G2 phase in the KPNA^KD^ cells vs. KPNA^pos^ (*p* = 0.040). Therefore, our results allow us to conclude for a cell cycle arrest in upon KPNA silencing also in the GBM cell line U87 MG. Potential sources underlying the above mentioned phase arrest may be related to the aberrant trafficking of cell cycle regulators between nucleus and cytoplasm. Indeed, KPNA2 has been already linked to the transport of a number of cyclins, such as p21 and Nijmegen B Syndrome Protein 1 (NBS1) [11]. Furthermore, the nuclear expression of KPNA2 correlated with those of NBS1 in a large series of malignant astrocytomas [8]. In addition, Huang *et al.* concluded that KPNA2 promoted tumorigenicity in epithelial ovarian carcinoma through up-regulation of the cell cycle related protein c-Myc [16].

Apart from its relation to cell cycle function c-Myc has been already identified as a key oncogenic transcription factor in a number of tumours [16], among others also in GBMs [23, 25]. C-Myc depends on the nuclear transporter protein KPNA2 to be translocated into the nucleus, where it is activated and promotes transcription of certain genes, responsible for tumour progression. To further gain insights into the role of KPNA2 in the regulation of c-Myc also in GBM cell lines, we analysed not only the total cellular expression but also the subcellular (nuclear vs. cytoplasmic) localisation of c-Myc before and after silencing of KPNA2. Although, we are aware of existing genetic and chromosomal events leading to an up-regulation of c-Myc in GBM [38, 39], silencing of KPNA2 demonstrated no effect on the total cellular expression of c-Myc. However, a more cautious analysis of its subcellular localisation revealed a strong trend (*p* = 0.062) for an accumulation of c-Myc in the cytoplasm, possibly eventuated as a consequence of an inhibition of the nuclear import of c-Myc upon KPNA2 knock down. Our results, provide further evidence for the promoting role of KPNA2 in the gliomagenesis, this time viewed from the aspect of up-regulation of the active translocation of oncogenic transcription factors into the nucleus.

The oncogenic contribution of KPNA2 in the pathogenesis of GBMs is further supported by the involvement of KPNA2 in an additional pathway, namely that of apoptosis. In general, inactivation of apoptosis has been already identified as a critical process for the tumorigenesis in several tumours [1]. KPNA2 is supposed to inhibit apoptosis in order to promote growth and survival of tumour cells as silencing of KPNA2 induced apoptosis in breast and bladder cancer cells as well as hepatocellular carcinoma cells, respectively [2, 15, 36]. In accordance with the literature, our results confirmed the anti-apoptotic role of KPNA2 also in the GBM U87MG cell line. After suppression of KPNA2 a significant inhibition of the anti-apoptotic protein bcl-2 as well an increased expression of the pro-apoptotic protein caspase-3 have been observed.

We will readily acknowledge several limitations of the present study. Our data are based on the analysis of four GBM cell lines; studies on animal models have not been conducted. Furthermore, we have not carried out any analysis of gene expression to reveal potential KPNA-regulated pathways and their putative alterations in GBM. As shown before [1, 8, 12] the family of karyopherins has been implicated in drug resistance in cancer; this effect of KPNA2 on chemosensitivity of GBM has not been investigated in the present study. Noteworthy, we will focus on this points in our forthcoming research.

Despite limitations, the current study confirms also in *in vitro* GBM models that a high expression of KPNA2 is associated with a more malignant phenotype. While increased expression of KPNA2 in human GBM cells promotes proliferation, invasion and aggressiveness of the tumorigenic cell pattern, silencing of KPNA2 displayed a less malignant profile of U87 MG cells. In particular, suppression of KPNA2 conferred an anti-proliferative behaviour, induced cell cycle arrest, facilitated cellular apoptosis and showed a strong trend for an inhibited nuclear import and the subsequent activation of the oncogenic transcription factor c-Myc. Our results strongly suggest that silencing of KPNA2 might be taken into account when designing new tailored therapeutic approaches for GBM, which remains still an incurable brain cancer.

## MATERIALS AND METHODS

### Cell cultures

The human glioma cell lines U87 MG, U373 MG, U138 MG and U118 MG are a kind donation of Prof. Dr. Scheffler of the Institute “Life & Brain” in Bonn. All cell lines are derived from tumours, which have been confirmed histopathologically as astrocytomas of the WHO grade IV (glioblastoma multiforme). The cell line U87 MG is hypoploide and stems from a 44 year old woman, the U138 MG hyperdiploide from a 47 year old man, while the U-118MG line is derived from a 50 year old and pentaploid. Together all the used tumour cell lines cover various genetical properties of different gliomas. The human astroglia cell line SVGp12 (ATCC CRL-8621) will serve as control cell line. The cells grow in adherent or 3D cell cultures under the addition of Dulbecco’s Modified Eagle Medium (DMEM) with 10% Fetal calf serum (FCS), 1% Penicillin-Streptomycin and 1% L-Gluatmin. Experiments were performed under sterile conditions in the own laboratories (Supplementary Table 2).

### Flow cytometric detection of KPNA2

To define the relative expression levels of the different glioblastoma cell lines (U87 MG, U118 MG, U373 MG, U138 MG), cells were harvested with trypsin (Gibco), washed with FACS buffer (PBS w/o Mg^2+^/Ca^2+^, 5% Bovine Serum Albumin, 0,5M EDTA) and the single cell suspension adjusted to 1 × 10^6^ cells/mL. Subsequently they were fixed with eBioscience Fixation Buffer according to the manufacturer’s protocol, stained for 30 min with unconjugated anti-KPNA2 (see Supplementary Table 1) and diluted in permeabliziation buffer. Fluorescence detection was obtained with FITC conjugated donkey anti-goat IgG (BD Bioscience) for 30 min. Expression levels were determined via flow cytometry (BD Calibur). Obtained data is analysed with FlowJo V10.1 and statistics calculated in Prism 5.0d.

### Silencing of KPNA2

The expression of KPNA2 is silenced by siRNA interference according to established protocols in human lung carcinoma cells [19]. A higher efficiency of the transfection was achieved by the use of “HiPerFect Transfection Reagent” following the manufacturer’s protocol. The negative control was performed with scrambled siRNA control. Knockdown efficiency was evaluated via immunofluorescence staining comparing silenced with non-silenced KPNA2.

### Proliferation assays

To determine the proliferation status of the U87 MG line, harvested cells are counted and dyed with a carboxy-fluorescein succinimidyl ester dye (BD Bioscience; CFSE-dye) according to the manufacturer’s protocol. Proper staining is evaluated via FACS analysis. The rest is seeded out in a density of 1 × 10^5^ cells/ well in a 24 well plate. After firm adherence of the cells (24 h after staining), silencing of KPNA2 is performed as described above. Proliferation rates are determined via FACS (BD FACS Calibur™; BD Bioscience) after 48 and 72 h of culturing post siRNA interference. Obtained data is analysed with FlowJo V10.1 and statistics calculated in Prism 5.0d.

### Methylthiazole tetrazolium (MTT) viability assays

To determine the proliferation status of the U87 MG line (KPNA2^KD^ vs. KPNA2^pos^ cells), cells are plated on a flat-bottom 96-well plate at a density of 1 × 10^5^ cells/well, knockdown performed and incubated at 37° C for 48 h. After the incubation period, MTT-Assay was performed according to manufacturer’s protocol (Roche; Cell proliferation kit I) and analysed with a BioTek microplate reader at a wavelength of 570 nm. Obtained data was exported to Excel V10.1 and statistics calculated in Prism 5.0d.

### Cell cycle analysis

Cells of the U87 MG are harvested adjusted in a single cell suspension to a cell count of 1 × 10^6^ cells/mL, fixed with 2% paraformaldehyde and subsequently stained with Propidium Iodide (Invitrogen™; 1,0 mg/mL). The DNA-intercalating fluorescence dye stains the whole cell population but according to the cells DNA content with different fluorescence intensities. DNA-content of the single cell suspension is determined via FACS (BD FACSCalibur™; BD Bioscience) analysis and obtained data evaluated with FlowJo 10.1. Statistics are calculated with Prism 5.0d.

### Immunofluorescence

Tumour cell lines, grown in monolayers of 60–80% confluency, were fixed with 4% PFA (Sigma Aldrich). Fixed cells were stained for KPNA2, different Transcription Factors, Ki67 and Bcl-2 (see Supplementary Table 1). All KPNA2 containing samples were stained with the secondary antibody Donkey-anti goat Alexa Fluor 488; TFs were stained with the secondary antibody goat-anti rabbit (see Supplementary Table 1 online), counterstained with DAPI and examined on an AxioImager.Z2 (Carl Zeiss) using 10× (numerical aperture 0.3) and 50× (numerical aperture 0.75) Plan-Neofluar magnification objectives. Images were analysed with AxioVision software (version 4.8; Carl Zeiss).

### mRNA isolation and quantification

RNA was isolated using the miRNeasy mini kit (Qiagen; Hilden; Germany). Complementary DNA was reverse transcribed using the Taqman Reverse Transcription Reagents (Life Technologies). Quantitative RT-PCR reactions were performed in triplicates using SYBR Select Master Mix (Life Technologies), respective primers (MWG; Supplementary Table 1) on a QuantStudio 7 real-time PCR system (Life Technologies). Data was normalized to Gapdh endogenous control.

All reagents and kits were used at manufacturer’s recommendation, if not stated otherwise.

### Western blot analysis

#### Sample preparation

1 × 10^6^ cells/mL of KPNA2^pos^ and KPNA2^KD^ cells were washed in phosphate-buffered saline (PBS), cells were pelleted by centrifugation and re-suspended in 100 µL radioimmunoprecipitation assay buffer (RIPA-buffer) (Cell Signaling Technology) with addition of Halt protease inhibitor cocktail (Thermo Scientific, Rockford, IL).

The cells were frozen and thawed, debris was removed by centrifugation for 8 min at 400g. Lysates were stored at –70° C until used. For SDS gel electrophoresis, samples were diluted directly into 4x SDS-gel sample buffer.(Laemmli Buffer).

#### Gel electrophoresis

Probes were subjected to standard SDS gel electrophoresis and Western blotting. SDS gel electrophoresis was performed in 10% or 12% acrylamide gels as previously described using Peqlab running chambers and semi-dry transfer system. Proteins were detected applying goat anti-KPNA2 (Santa-Cruz; 1:75), mouse anti-beta actin (Cell Signaling Technology; 1:500), mouse anti-Caspase 3 (Santa Cruz; 1:500), mouse anti-Caspase 8 (Santa Cruz; 1:500), mouse anti-Bcl2 (Santa Cruz; 1:500), mouse anti-c-MYC (Santa Cruz; 1:500) antibodies. Visualization was achieved via chemilumineszenz using the clarity ECL solution (BioRad) and an PeqLab Fusion imaging system (both LI-COR Biosciences, Lincoln, NE) scanner. Protein quantifications were performed using

The Odyssey software. The intensity for each band was determined and normalized to the intensity of the actin band of the corresponding probe.

### Statistical analysis

Statistical analysis was performed using GraphPad Prism for Macintosh, version 5.0d. Statistical significance of immunofluorescence data was determined using the two-tailed unpaired Student’s *t*-test and differences were considered significant at a *P*-value below 0.05.

Other statistical analyses (cell cycle analysis; proliferation assay) were performed using the one-way ANOVA combined with Bonferroni’s multiple comparison test.

## SUPPLEMENTARY MATERIALS FIGURE AND TABLES


