# Supplementary material for: Silencing of the nucleocytoplasmic shuttling protein karyopherin a2 promotes cell-cycle arrest and apoptosis in glioblastoma multiforme
Source: Oncotarget. 2018 Sep 11;9(71):33471–81. doi: 10.18632/oncotarget.26033 (PMC6173355; doi:10.18632/oncotarget.26033)
Supplement: Supplementary file 1 [file oncotarget-09-33471-s001.pdf]

Silencing of the nucleocytoplasmic shuttling protein karyopherin a2 promotes cell-cycle arrest and apoptosis in glioblastoma multiforme

SUPPLEMENTARY MATERIALS

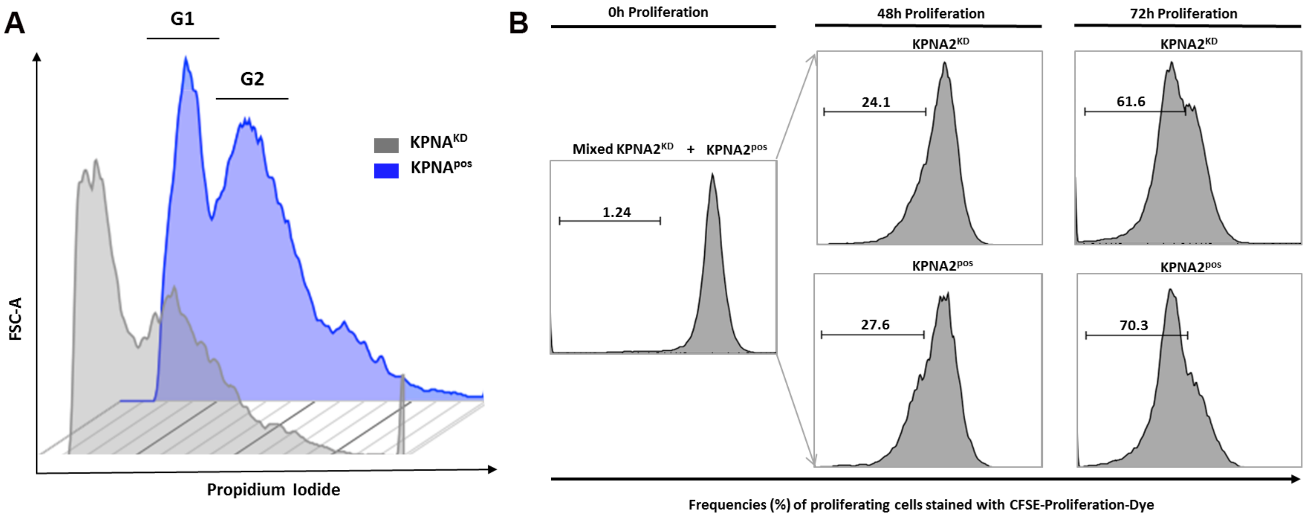

Supplementary Figure 1: (**A**) Original flow cytometry data of the cell cycle analysis conducted via Propidium Iodid (PI) staining, (**B**) Histograms of the CFSE-proliferation assay.

Supplementary Table 1: Primary and secondary antibodies used for immunofluorescence

Antibody	Host	Dilution	Source
KPNA2	Goat	1:75	sc6917; santa cruz
c-Myc	Rabbit	1:100	CS13987S; Cell Signaling
NFKb	Rabbit	1:100	CS13586S; Cell Signaling
Oct-4	Rabbit	1:100	CS2840S; Cell Signaling
Ki67	Mouse	1:75	14-5698-82; eBioscience
Bcl-2	Mouse	1:75	562529; BD Bioscience
b-Tubulin	Mouse	1:100	556321; BD Bioscience
Alexa Fluor 488 donkey anti mouse IgG	Goat	1:500	A21202; Invitrogen
Alexa Fluor 488 donkey anti goat IgG	Donkey	1:500	A21208; Invitrogen
Alexa Fluor 555 donkey anti rabbit IgG	Rabbit	1:500	A31572; Invitrogen
FITC donkey anti-goat IgG	Donkey	1:200	BD Bioscience

Supplementary Table 2: Primer for quantitative Real-Time PCR on SYBR Green basis

Primer	Sequence
Caspase 3	Fwd: 5′ TTGTGGAATTGATGCGTGT 3′<br>Rev: 5′ GGCTCAGAAGCACACAAACA 3′
Caspase 8	Fwd: 5′ CTTCGTGCCTGCCTGTACC 3′<br>Rev: 5′ CGFGCCCAGAAAGTGGACG 3′
GAPDH	Fwd: 5′ ACCACAGTCCATGCCATCAC 3′<br>Rev: 5′ TCCACCACCCTGTTGCTGTA 3′
